# Designing a doctor evaluation index system for an online medical platform based on the information system success model in China

**DOI:** 10.3389/fpubh.2023.1185036

**Published:** 2023-10-12

**Authors:** Shuaibing Liu, Yunqiu Zhang

**Affiliations:** Department of Medical Informatics, School of Public Health, Jilin University, Changchun, China

**Keywords:** doctor evaluation, online medical platform, information system success model, Delphi, service quality

## Abstract

**Objective:**

In the context of “internet + medical health” and emphasis on evaluation mechanism for medical and health talents in China, we design an evaluation index system for doctors on online medical platforms by synthesizing two patterns of existing online medical platforms, which is the first step to enhance the capabilities of doctors on online medical platforms.

**Methods:**

Based on the doctor evaluation model integrating information systems success model (ISS-DE model) and grounded theory, the evaluation indicators were obtained through expert interviews, offline medical institutions investigation, online platforms investigation, and literature research, and were assigned weights using the analytic hierarchy process (AHP) method. A working group composed of 23 experts was set up to review and determine the competency standards of doctors on the online medical platforms.

**Results:**

A new indicator framework covering 3 dimensions of system quality, service quality and information quality was constructed in this study. The index system included 3 first-level indicators, 8 s-level indicators and 60 third-level indicators, and each indicator was given different weightage.

**Conclusion:**

The complete index system constructed by the Delphi method in this study is suitable for China’s online medical platforms, which will help to improve the quality of platforms and the ability of doctors, thus promoting the process of internet medical integration.

## 1. Introduction

In recent years, with the penetration of mobile internet technology in all walks of life and the gradual improvement in people’s health awareness, online medical platforms, as the product of the interpenetration of the internet and the medical and health industry, have brought together quantities of medical and health information (1). On April 25, 2018, the State Council issued the “*Opinions on Promoting the Development of Internet + Medical Health*,” where two patterns of online medical platforms have been proposed: medical institutions as the main body (representative hospitals include Peking University First Hospital, West China Hospital, Sichuan University, etc.) and internet companies and enterprises as the main body (often referred to as online health community, OHC), respectively (2). The spread of COVID-19 boosted the development of online medical platforms in China to a considerable degree in terms of its scale and variety. According to China Internet Network Information Center (CNNIC’s) *50th Statistical Report on the Development Status of the Internet in China*, as of June 2022, the scale of online medical users in China reached 300 million, accounting for 28.5% of internet users as a whole (3).

The increasingly advanced technology has enhanced the development of online medical platforms. Nevertheless, the doctors still have been the core role in the process of online medical care, and the qualifications of doctors are necessary to be evaluated in order to establish the admission criteria of the platforms and ensure the treatment effect on the patients (4). Talent evaluation is an important part of the talent development system and mechanism, as well as a prerequisite for the management and use of talent resources (5). Many policy documents emphasize the establishment and improvement of a evaluation mechanism for medical and health talents (6–8), but the current evaluation mechanism for online medical platform doctors in China still has problems such as imperfect evaluation standards, relatively lagging professional construction of evaluation teams and incomplete information utilization (9), which may affect the scientificity of platform doctors’ evaluation and cause certain obstacles to the incentive of platform doctors. It is urgent to solve these problems by ameliorating the evaluation index system of online medical platform doctors.

The construction of an evaluation index system lays the basis for solving the evaluation problems of online medical platforms doctors (10). The existing doctor evaluation system, with only one single application scenario, still focuses on the performance evaluation of doctors in offline hospitals (11–15) and the application of related evaluation indicators (16–19). The information technology represented by the internet has broken the barriers between online and offline medical treatment, providing a new platform for doctors to practice medicine. However, the existing studies mainly focus on the online medical platforms, involving the construction of information service quality evaluation index system (20–22), and the discussion on the influencing factors of user satisfaction (23). But for doctors, the core of medical service, have not been manifested completely and in-depth in the existing evaluation systems. Furthermore, the comment text of the platforms is the solely data source of almost existing evaluation systems, where behavioral characteristics indicators and doctor subject attributes indicators are ignored, but only textual characteristics are considered. In this way, some biases on the evaluation results might be caused to impact the effect of evaluation systems. Recently, there are several studies indeed have shifted into the issue of doctor evaluation in the online medical platforms (24–26). However, such studies have been conducted from the perspective of patients and only focused on a single type of online medical platform, and the comprehensiveness of the indicators was inadequate.

Most previous studies focused on the influencing factors or the research objects were doctors in offline physical hospitals, but few studies build a comprehensive evaluation index system for doctors in online medical platforms. As the core of online medical treatment, doctors are required to have more comprehensive and targeted standards to evaluate and measure them in this context. Therefore, this study aims to construct a multi-source and multi-dimensional online medical platform doctor evaluation system, with the adoption of rigorous research methods and the integration of multiple online medical platforms, in order to evaluate doctors in online platforms comprehensively and systematically.

## 2. Theoretical basis

The online medical platform is a one-stop service platform that relies on physical hospitals to provide online follow-up and routine consultations. Considered from a systems perspective, online medical platforms are essentially computer-supported web-based information systems (27). The process of doctors providing information and services on the online medical platforms conforms to the operation mode of the information system (IS). The DeLone and McLean IS success model was developed to measure the success of ISs from a cause-and-effect perspective. Its validity and usefulness have been demonstrated in the field of evaluating the success of IS through numerous empirical studies (28–30), and it is also widely used in the field of healthcare (27, 30). Therefore, this paper adopts the IS success model as the basis for constructing the doctor evaluation system of online medical platforms.

### 2.1. Information system success model

**Figure 1:**
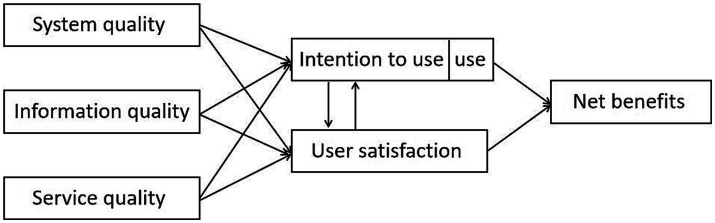
DeLone and McLean updated IS success model.

The IS success model was first proposed by DeLone and McLean in 1992 (31) and optimized in 2003 (32). The model shows the basic structure of the IS, which consists system quality, information quality, service quality, intention to use (use), user satisfaction, net benefits, and the relationship among the six essential factors. Figure 1 shows the updated model.

In order to apply the IS success model into the field of doctor evaluation more flexibly, we propose a modified DeLone and McLean IS success model, named IS success-doctor evaluation model (ISS-DE model) and defines the essential factors of ISS-DE model in detail. Doctors, as the subject of IS, provide information and services to online platform patients, so doctor evaluation dimensions correspond to system quality, service quality and information quality of the model. The patients’ intention to choose a doctor/choose a doctor corresponds to the intention to use/use in the model and the patient satisfaction corresponds to the user satisfaction.

**Figure 2:**
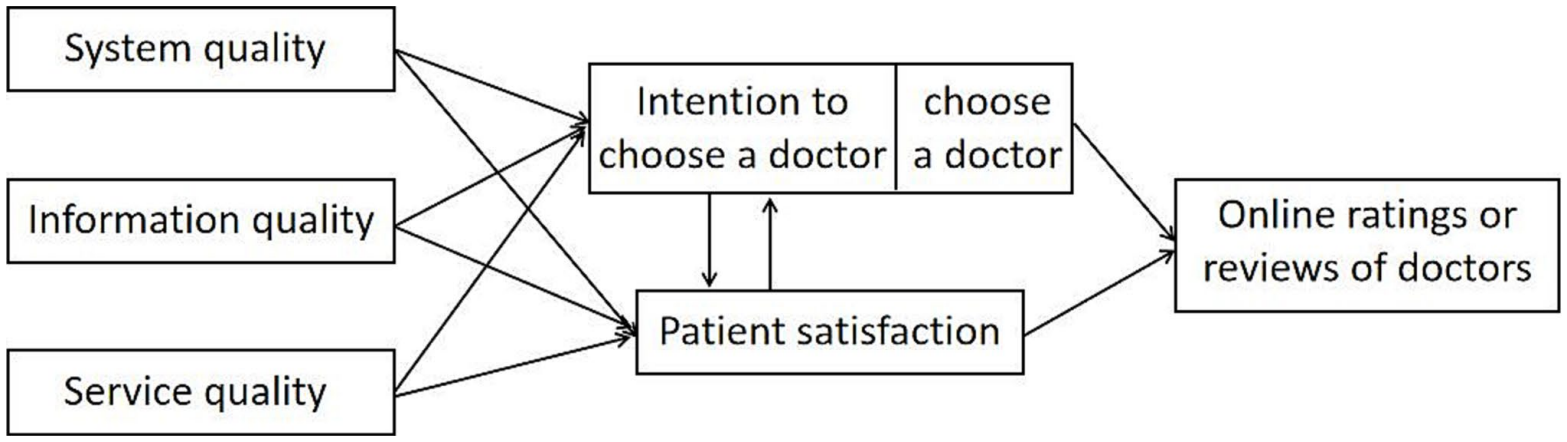
ISS-DE model.

Net benefits is an indicator to judge the success of the information system, whose value and significance in the application of IS success model to practical research has gradually diminished with the continuous development of the internet. And many current studies have removed the variable of net benefits and took use or user satisfaction as the dimensions used to evaluate the success of ISs (33–35). However, no matter how to improve IS success model, it should have variables that can reflect the benefits, impacts or outcomes of the IS. Therefore, in this paper, three indicators, patient reviews/ratings for doctors, the patients’ intention to choose a doctor/choose a doctor and patient satisfaction, are used to assess doctor competence instead of only net benefits. In summary, Figure 2 shows the ISS-DE model.

Based on the ISS-DE model, this study proposes that online medical platform doctor evaluation involves three dimensions: system quality, information quality, and service quality. System quality is a measure of an IS’s own characteristics (36), while the research object of this study is platform doctors. Therefore, system quality is used to evaluate the characteristics of doctors’ main attributes, including type of doctors and influence of doctors. Information quality is a related evaluation of whether the content and utility of health information provided by doctors on the platform can meet the needs of patients (37). It consists of two dimensions based on common practice in existing research (38): information content and information utility. Service quality is the users’ evaluation of the service obtained from the IS (39). By synthesizing the existing service quality models and related theories of evaluation, this paper constructs a doctor service quality evaluation model named technology-function-procedure-value model (TFPV model). Among them, technical quality and functional quality come from the service quality model proposed by Grönroos, and technical quality reflects the technical ability of doctors and the accuracy of diagnosis and treatment (25, 40); functional quality is patients’ perception of doctors’ service attitude and whether doctors adhere to professional standards; procedure quality is derived from the empathy dimension of the SERVQUAL model, reflecting whether doctors care about and provide personalized services to platform users (41); value quality is proposed based on the theory of perceived value and fairness, reflecting patients’ evaluation of the rationality of charges and price differences for different services provided by doctors (42).

## 3. Materials and methods

**Figure 3:**
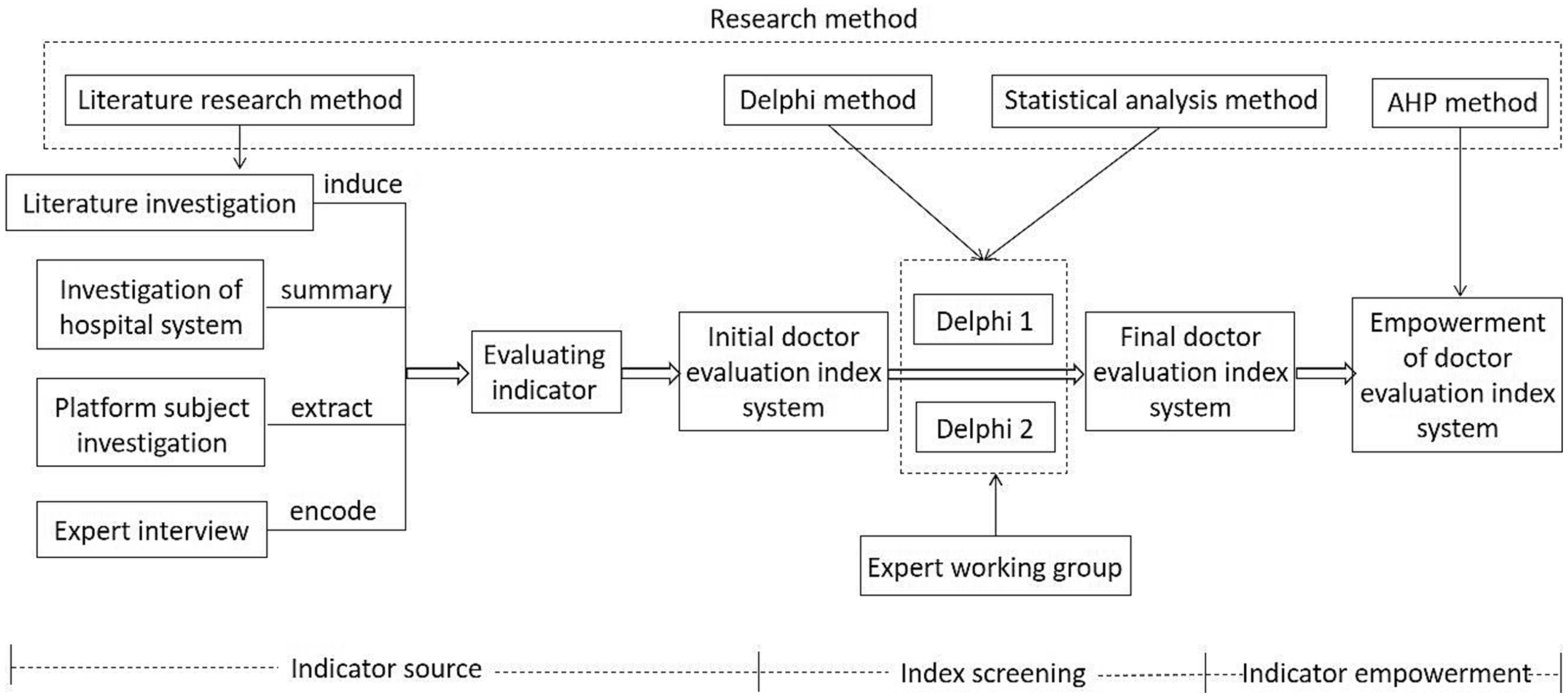
Flow chart of doctor evaluation research design of online medical platform.

Our study takes three steps to construct the doctor evaluation index system of online medical platforms. In order to solve the drawbacks of a single data source and application scenario, we obtain multi-source indicators from four aspects based on the principle of integrity, and then correspond these indicators to the dimensions of the ISS-DE model. In addition, this study combines the perspectives of patients, doctors and hospitals/platforms managers, which makes up for deficiency of single research perspective in previous studies. Based on the consideration of research objects and data types, the paper selects Delphi method and AHP method to construct evaluation index system. An expert group is invited to select the indicators through two rounds of Delphi screening of the initially constructed indicator system. Finally, the AHP was used to give weight to the index system, and the comprehensive weight of each indicator was defined. Figure 3 shows the research and design process of this study.

### 3.1. Indicator source

For the purpose of widely obtaining evaluation indicators, the study fully considered the source of indicators and adopted four methods: expert interviews, offline medical institutions investigation, online platforms investigation, and literature research to obtain indicators that correspond to the model.

First, the semi-structured interview method is used to collect data, and the evaluation indicator was extracted by coding the text of the expert interviews. Table 1 shows examples of indicators extraction from original interview text.

**Table 1:** Index extraction of interview text.

Variable induction	Initial coding	Original statement example
System quality	Doctors’ resume	I think there should be a summary of the doctors’ achievements; doctors’ resume, including study and further education, personal achievements, and areas of expertise
	Peer review	Peer review and the amount of forwarding can be considered
	Doctor activity	Doctors are not enthusiastic about participating
	Patient satisfaction	Patient evaluation, patient favourable comment rate, complaints, and disputes
	Patient support	The popularity of publishing articles, collection number, likes number, cited frequency
Information quality	Information specialty	From popular science information, we can see whether his thinking, diagnosis, and treatment level are reliable. We should start with the doctors’ professionalism and evaluate the doctor regularly
	Information correlation	Relevance between content and question.
	Information intelligibility	Try to use technical terms as little as possible, or explain terms in plain language so that ordinary patients can understand them
	Information usefulness	The size of help to patients
Service quality	Communication ability	Online examination and communication skills should be evaluated, as should communication skills with patients
	Medical ethics	Doctors’ professional level, communication ability, and humanistic care
	Service pertinence	A template is often used as a reply. Standard replies fail to comprehensively consider the actual situation of patients

In the research of offline medical institutions, this paper investigates the performance evaluation standards and indicators of multiple offline medical institutions, such as service attitude and service skills. As for the online medical platforms, the investigation is based on the top 100 internet hospitals in 2020 published by Internet Weekly (43) and the Development Report of Internet Hospitals in China in 2021, jointly published by the National Center for Telemedicine and Internet Medicine and the health sector (44). By investigating 28 online medical platforms with medical institutions as the main body and 39 with internet companies as the main body, 78 indicators related to doctor evaluation have been extracted.

In terms of literature research, the keywords including “doctor evaluation, “doctor selection”, “patient satisfaction”, “information quality”, and “service quality” are used to conduct a systematic literature search in the electronic database. Additionally, under the guidance of the index framework, the national practice guide “*General Practitioners*” *Professional Technical Ability Evaluation Guide* is reviewed to extract candidate indicators. The final version of indicators collected and established through four methods have been synthesized and matched with the corresponding dimensions in the ISS-DE model.

### 3.2. Indicator screening

The Delphi method is a common method for constructing index system, and it is also the most important decision-making index for identification and screening (45). It is a method that requires the collective judgment of experts in related fields (46), generally being required 10–15 experts being required to obtain the desired results (47).

#### 3.2.1. Expert selection

The basic criteria for our research and selection of experts include (i) expert authority, having an online medical platform account operated by themselves, or having experience in using online medical platforms; (ii) wide functions, including clinical medical personnel, scientific research management staff, hospital administrators, and internet office staff; (iii) expert qualifications: a bachelor’s degree or higher is compulsory. Besides, experts should have intermediate title or above, or be very familiar with online medical platforms.

#### 3.2.2. Questionnaire composition

The Delphi expert questionnaire was designed for the preliminary evaluation index system of doctors on the online medical platform. The questionnaire included: (i) an invitation letter for experts, including research purpose, research background, and instructions for filling in the form; (ii) basic information on the experts, including gender, age, education, occupation, unit, professional title, online medical platform used, duration of use, and experts’ scores on their familiarity and judgment basis in this field; and (iii) an indicator evaluation table, including the scores of various indicators and a comments column. Experts rated the importance and relevance of doctors’ evaluation indicators on the online medical platform. The scoring standard was a 5-point Likert scale, and the scores were set from 1 to 5, representing “very unimportant”, “unimportant”, “average”, “important” and “very important” respectively. Moreover, experts could post problems and suggestions for corresponding indicators in the comments column.

#### 3.2.3. Data analysis

The scientific rationality of the Delphi method is reflected in the effective response rate of experts, authority coefficient (*Cr*), and coordination coefficient.

1) The effective response rate of experts determines the credibility and scientific basis of the results. Authoritative data show that the effective response rate of 50% is the lowest acceptable value of the Delphi method, 60% is considered medium, and more than 70% indicates a very good standard (48).
2) The expert authority coefficient is generally determined by two factors: the judgment coefficient (*Ca*) represents the evidence of the expert’s judgment; and the familiarity coefficient (*Cs*) represents the expert’s familiarity with the problem. *Cr* can be calculated by the formula $Cr=\left(Ca+Cs\right)÷2$ . Usually, the expert *Cr* ≥ 0.7 indicates that the experts selected in this survey have good authority.
3) Coordination coefficient: Kendall’s *W* consistency coefficient test is used to evaluate the quality of expert consultation and measure the difference in importance, feasibility, and sensitivity of expert opinions for each indicator. The statistical significance of Kendall’s *W* test results shows that experts have reached a consensus.

In addition to judging the Delphi quality from the above aspects, the calculation methods of each indicator score included: (1) the arithmetic mean of each indicator score; (2) full mark rate; and (3) coefficient of variation (CV), reflecting the fluctuation degree of experts’ scores on various indicators. The smaller the CV value, the more concentrated the opinions of experts on this indicator. (4) critical value of average value, Thecriticalvalue=Mean−Standarddeviation , and the score above the critical value is selected; (5) critical value of full mark rate, TheCriticalValue=Fullmarkrate−Standarddeviation , and the score above the critical value is selected; (6) critical value of the CV, which is TheCriticalValue=CV+Standarddeviation , and the score below the critical value is selected.

### 3.3. Indicator empowerment

AHP was used to assign weights to the evaluation indexes of online medical platform doctors. At present, the most widely used AHP method is to compare the judgment matrix with the importance average (49). Using the Saaty1–9 scale method, after comparing the average of importance assignment of each indicator at the same level, the corresponding judgment matrix was constructed (50, 51).

In the AHP, if the matrix order is more than 2, it will affect the consistency result of the judgment matrix, so a consistency test is needed. The consistency index (CI) can judge the effect of the consistency test. However, CI alone cannot determine the consistency test results. Therefore, some scholars have introduced the average random consistency index (RI). The result of consistency judgment is determined by calculating the consistency ratio (CR, $CR=\frac{CI}{RI}$ ). It is generally accepted that CR < 0.1 proves that the error value is small and the overall consistency is good.

## 4. Results

### 4.1. Basic information of experts

A total of 23 experts with specific experience from 10 representative hospitals in six cities, including Shenzhen, Hangzhou, Changchun, Shenyang, Wuhan, and Changzhou, were invited to participate in two rounds of expert consultation. We have collected the personal information of experts (Table 2). The effective recovery rates of two rounds of expert consultation questionnaires are 82.61% and 91.30%, respectively, showing that experts were highly involved.

**Table 2:** Basic information of experts.

Category		The first round (*N* = 19)		The second round (*N* = 19)	
		Number of people	Constituent ratio (%)	Number of people	Constituent ratio (%)
Gender	Men	12	63.2	12	57.1
	Women	7	36.8	9	42.9
Age	30–40 years old	4	21.1	5	23.8
	41–50 years old	15	78.9	16	76.2
Degree of education	Doctor and above	5	26.3	6	28.6
	Master	10	52.6	11	52.4
	Bachelor	4	21.1	4	19.0
Professional title	Senior title	8	42.1	7	33.3
	Vice-senior title	5	26.3	5	23.8
	Intermediary title	5	26.3	8	38.1
	Junior title	1	5.3	1	4.8

The degree of expert authority reflects the reliability of the survey. The degree of expert authority *Cr*_1_ in the first round of the questionnaire is 0.805. The expert authority *Cr*_2_ in the second round of the questionnaire is 0.874. The *Cr* of the two rounds of expert questionnaires is greater than 0.7, which proves that the experts have a high degree of authority, and their opinions could be used as the basis for selecting the evaluation indicators of doctors on the online medical platforms.

### 4.2. Online medical platform doctor evaluation results of Delphi

In the first round of expert consultation, the critical value method is used to select indicators. In order to build a comprehensive evaluation index system and ensure that important indicators are not excluded, only indicators that did not meet the above three measurement criteria are excluded. If only two measurement criteria are not satisfied, such items will be added in the second round of questionnaire to ask the experts if they agree to delete these indicators. Of the 69 items evaluated by experts in the first round, one item is immediately rejected for inclusion in the list; five items are set as expert inquiry items in the second round. Then, experts are asked whether they agree to exclude these items, and the final agreed exclusion rate will be calculated. And if the agreed exclusion rate of the inquiry item is more than 50%, it will not be included in the index system. At the end, a total of 63 items directly enter the second round of expert consultation.

**Figure 4:**
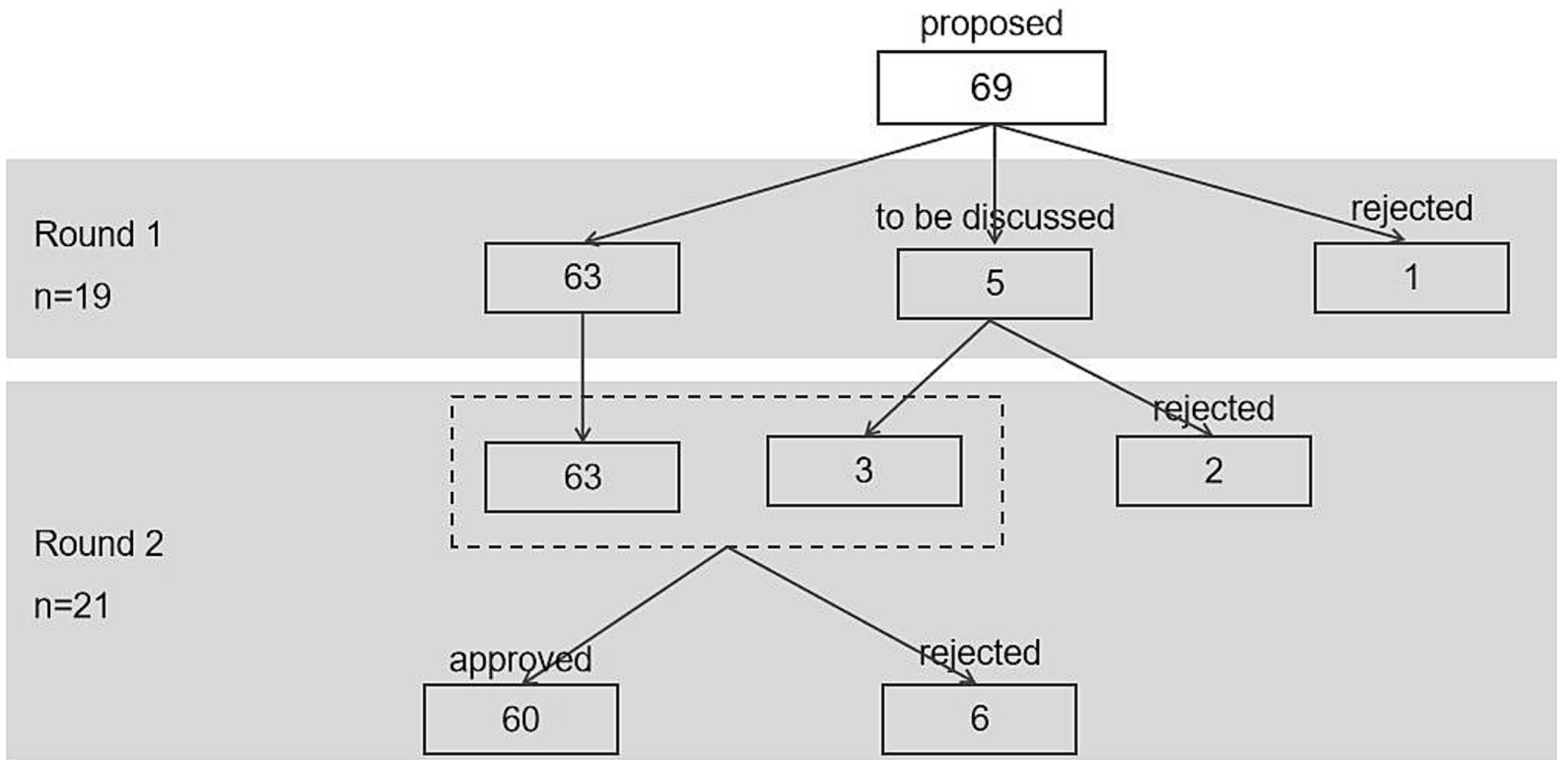
Flow chart of approval/rejection of two rounds of project expert evaluation.

In the second round of consultation, the indicator screening criteria are improved, and another new exclusion criteria are included based on the threshold method. For two indicators whose measurement criteria do not meet the threshold method, the average value is >3.5 and the CV is <0.25 as the new screening criteria (52). In the second round of expert evaluation, the agreed exclusion rates of the two indicators are 52.4% and 71.4%, exceeding 50%, so they are both excluded. After the expert evaluation, the remaining 3 items and the 63 items in the first round are jointly evaluated in the second round, 60 items are approved for inclusion in the final list, and 6 items are rejected. Figure 4 shows the indicator screening process.

### 4.3. Consistency test of expert survey results

SPSS 25.0 is used to perform a statistical analysis of the Kendall coordination coefficient on the importance score of evaluation indicators in the consultation form. Table 3 shows the Kendall analysis results. The results demonstrate that the two rounds of consultation are significant (*p* < 0.05), the experts’ scores are consistent, and the evaluation results are reliable.

**Table 3:** Kendall coordination coefficient.

	Round 1			Round 2		
	*W* value	*X*^2^ value	*p*-value	*W* value	*X*^2^ value	*p*-value
First-level indicators	0.33	13.857	0.001^**^	0.175	7.366	0.025^*^
Second-level indicators	0.176	36.865	0.000^**^	0.165	34.668	0.000^**^
Third-level indicators	0.185	239.070	0.000^**^	0.226	317.964	0.000^**^

^**^p < 0.01, ^*^p < 0.05.

The overall coordination coefficient of the first round of expert consultation is 0.177; the overall coordination coefficient of the second round of expert consultation is 0.216; and the Kendall coordination coefficient of the second round of expert consultation is higher than that of the first round, suggesting that after the first round of consultation, the consensus of experts on the relative importance ranking of all indicators had been improved.

After two rounds of expert evaluation, the experts’ opinions are unified, and the degree of unity are testified to be good. The researchers of this paper have discussed the experts’ opinions, merged 3 s-level indicators, and deleted 9 third-level indicators. After completing all the procedures of the Delphi method, the final core evaluation framework includes 60 third-level indicators, corresponding to 8 s-level indicators, and maps on three first-level dimensions. (See Appendix 1 for the interpretation of the final index system and evaluation index.)

### 4.4. Determination of index weight results by the AHP method

**Figure 5:**
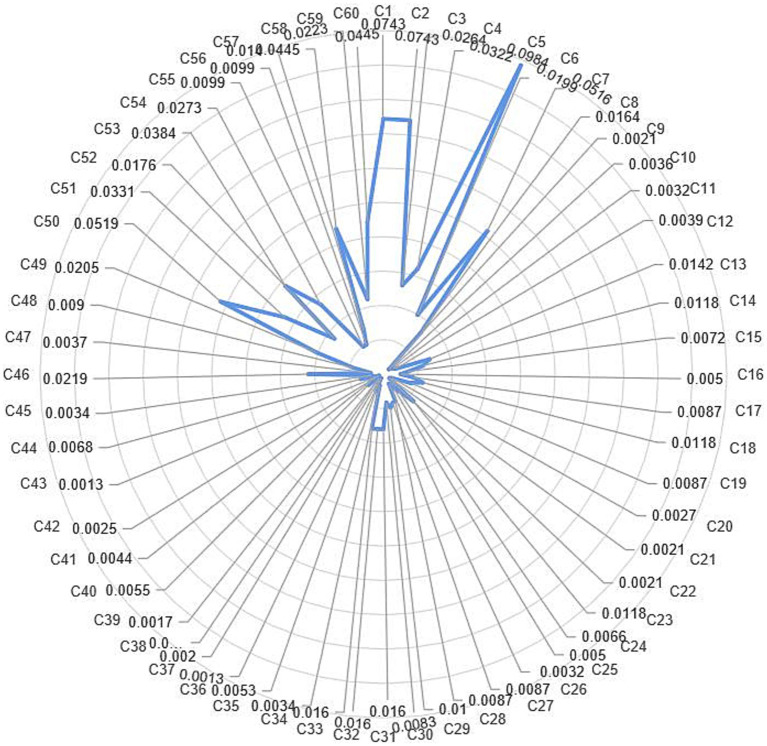
Doctor evaluation index system and weight value of online medical platform.

The reasonable degree of weight setting will affect the objectivity and fairness of the results. In this study, AHP has been used to distinguish the weights of evaluation indexes, and the hierarchical model and judgment matrix are established according to the evaluation index system. Finally, the index system weight table (Table 4) and index weight diagram (Figure 5) at all levels have been obtained.

**Table 4:** List of total weights of doctor evaluation index system of the online medical platform.

Project	Weight	Combination weight
**A1 System quality**	0.5247	—
B1 Type of doctors	0.75	0.3935
C1 The tier level of hospitals	0.1889	0.0743
C2 Doctors’ title	0.1889	0.0743
C3 Academic title	0.0670	0.0264
C4 Doctors’ education	0.0819	0.0322
C5 Working years	0.2501	0.0984
C6 Graduate school	0.0505	0.0199
C7 Work-results	0.1311	0.0516
C8 Teaching experience	0.0417	0.0164
B2 Influence of doctors	0.25	0.1312
C9 Reprint volume	0.0162	0.0021
C10 Number of likes	0.0277	0.0036
C11 Collection quantity	0.0243	0.0032
C12 Attention number	0.0300	0.0039
C13 Favorable rate	0.1086	0.0142
C14 Recommendation rate	0.0897	0.0118
C15 Adoption rate	0.0551	0.0072
C16 Number of patients	0.0381	0.0050
C17 Reserved quantity	0.0662	0.0087
C18 Page view	0.0897	0.0118
C19 Peer review	0.0662	0.0087
C20 Recommended degree	0.0209	0.0027
C21 Contribution value	0.0162	0.0021
C22 Is it “recommended”?	0.0162	0.0021
C23 Comprehensive score	0.0897	0.0118
C24 Doctor label	0.0504	0.0066
C25 Complaint	0.0381	0.0050
C26 Number of questions answered	0.0243	0.0032
C27 Follow-up rate	0.0662	0.0087
C28 Offline medical treatment rate	0.0662	0.0087
**A2 Service quality**	0.1416	
B3 Technical quality	0.5306	0.0751
C29 Basic theoretical knowledge of clinical medicine	0.1331	0.0100
C30 Basic pharmacological knowledge and clinical rational drug use knowledge	0.1100	0.0083
C31 Judgment and interpretation of common auxiliary examination	0.2136	0.0160
C32 Mastery and application of routine diagnosis and treatment operation technology	0.2136	0.0160
C33 Diagnosis and treatment of common and frequently-occurring diseases	0.2136	0.0160
C34 Majors are good at diseases	0.0457	0.0034
C35 Treatment experience	0.0705	0.0053
B4 Functional quality	0.1531	0.0217
C36 Interpersonal communication skills	0.0588	0.0013
C37 Patient-centered service concept	0.0929	0.0020
C38 Abides by professional ethics and behavioral ethics	0.1395	0.0030
C39 Can provide confidence for patients	0.0789	0.0017
C40 Confidentiality	0.2555	0.0055
C41 Service interactivity	0.2025	0.0044
C42 Interactive real-time	0.1130	0.0025
C43 Service friendliness	0.0588	0.0013
B5 Value quality	0.0718	0.0102
C44 Rationality of charges	0.6667	0.0068
C45 The price difference for different services provided by doctors	0.3333	0.0034
B6 Procedure quality	0.2445	0.0346
C46 Service content personalization	0.6333	0.0219
C47 Personalized service mode	0.1062	0.0037
C48 Service continuity	0.2605	0.0090
**A3 Information quality**	0.3338	
B7 Information content	0.6667	0.2225
C49 pertinence	0.0919	0.0205
C50 Professionality	0.2331	0.0519
C51 Accuracy	0.1485	0.0331
C52 Objectivity	0.0791	0.0176
C53 Integrity	0.1726	0.0384
C54 Comprehensibility	0.1225	0.0273
C55 Expression diversity	0.0447	0.0099
C56 Comprehensiveness	0.0447	0.0099
C57 Simplicity	0.0629	0.0140
B8 Information utility	0.3333	0.1113
C58 Validity	0.4000	0.0445
C59 Practicality	0.2000	0.0223
C60 Safety	0.4000	0.0445

## 5. Discussion

This study constructed an ISS-DM model based on the IS success model from three dimensions: system quality, service quality, and information quality. Compared with the patient satisfaction inclusive model constructed from the patients’ perspectives (53), this paper integrates the perspectives of patients, doctors and hospitals/platforms managers, and constructs a more comprehensive and complete index system.

1) First, system quality (52.47%) plays a leading role in the evaluation. In our study, system quality includes type of doctor (39.35%) and influence of doctor (13.12%), among which the number of working years, tier level of hospital and doctor’s title rank the top three. Previous research had found that doctors’ title and the tier level of hospitals have an impact on patients’ choices of doctors (54), indicating that the most important factor for doctors to improve their own evaluation is their educational backgrounds and professional titles. Doctors should continuously expand their clinical and teaching experience through continuous examination and evaluation to obtain corresponding results. It is worth mentioning that although work-results (5.16%) still ranks high in the whole index system, it has changed from a decisive indicator to an extra point, which coincides with the background of breaking the “paper-centric” (only pay attention to the paper, but ignoring any others) in doctor evaluation in China. Further, we find that the evaluation generated by patients, such as favorable rate, recommendation rate and page view, are important indicators affecting the evaluation results of doctors. This finding is consistent with previous research that positive subjective information steadily and positively influences patients’ offline decisions (55).
2) Information quality (33.38%) is ranked second in weight. At present, there is no clear universal standard for evaluating the quality of online medical information (56). A prior study categorized OHC information according to its sources (patient-generated and system-generated) and explored its effects on patients’ online searches, evaluations, and decisions (57). There are also studies classifying online medical platform information into objective information and subjective information (55). However, this classification is different from this study, where the information quality refers to the quality of the information provided by the doctors, rather than the comments of the doctors from patients in the platform and the information given to the doctors by the platform. From the results of the study, the information quality provided by doctors is weighted more than the service quality, contrary to the studies conducted from the patients’ perspective (58). In this dimension, the information content provided by doctors is more important than the information utility. The professionalism and accuracy of the information content are the essential indicators determining the quality of information.
3) Finally, service quality (14.16%) is ranked third in weight. Although service quality was considered the decisive factor in patient satisfaction research (24, 59), from the perspective of managers, service quality is less important than system and information quality. In the service quality dimension, the technical quality has the greatest weight. A previous study suggested that doctors with high technical quality and functional quality were more likely to attract patients (25). Patient safety is a worldwide problem and a basic requirement for medical services. Therefore, technical quality, directly affecting patient safety, is given top indicator in the evaluation of doctors service quality; Procedure quality, reflecting the degree of personalization of doctors’ services, ranks second. In the context of the rapid development of internet medicine, doctors can break the passivity of traditional medical services and provide personalized information and services according to the needs of users. In this process, the needs of patients can be accurately met, and patients’ trust is established by medical institutions and platforms, thus achieving a win-win situation between doctors and patients; Functional quality, the basis of doctor services, depends on the attitude of doctors and the process of service interaction. In this dimension, confidentiality, service interactivity, compliance with professional ethics and behavioral ethics are among the top three indicators. There is an unexpected finding that the importance of patient-centered service concept has decreased in the research from doctor perspective, which used to be the most important indicator in the patients-oriented studies (60); value quality is the least weighted in this dimension. Our results are consistent with (60), which demonstrate that price is not an important indicator of patient satisfaction. In the doctors’ opinion, the value quality during the diagnosis should not be a determining factor in evaluating doctors, although the price of medical services is the most direct and realistic interest of people.

The calculation results of the weight of online medical platform doctors’ evaluation index confirms that the key points of improving the evaluation level of doctors at present should be in the following aspects:

Based on self-empowerment, improve medical ability by accumulating clinical experience, produce scientific research results, and improve clinical skills and scientific research level jointly. The evaluation results of doctors’ academic qualifications and professional titles are still the “gold standard” of doctor evaluation. Doctors need to constantly improve their academic qualifications to get higher evaluation. Medical institutions should promote the diversified evaluation methods such as vocational qualification evaluation, professional skill level identification, special skill assessment and other diversified evaluation methods, so as to make the evaluation results can be organically interrelated with the actual ability of doctors.

In terms of information quality, doctors themselves should not only ensure the accuracy and integrity of the information provided but also take into account the safety and understandability of the information. Furthermore, they should be responsible for the information provided in the platform to prevent patients being misunderstood by scientific errors. Moreover, patients’ autonomy and informed privilege should be treated with respect. The online medical platform should establish a clear reward and punishment system, which should not only encourage doctors to pay attention to the quality of information content when serving users, but also timely deal with medical information errors and give feedback to patients. In addition, the online medical platform should strengthen inspection and screening when doctors release medical information, and repeatedly filter false medical information to ensure the authenticity and usefulness of medical information.

Have an outstanding service quality will be supplementary bonus for doctor evaluation. Only by improving the service level, improving patient satisfaction and pursuing patient loyalty, can the social and economic value of doctors be realized. Having a high level of medical technology, complete medical theoretical knowledge and rich clinical work experience, as well as the ability to independently solve or deal with the rescue of general difficult cases and critical cases, are the basic qualities and service core of doctors. On the other hand, the price of medical service is the most direct and realistic interest issue of the people. Medical institutions and online medical platforms should design and unify the charging standards, as well as set the reasonable charging prices. Meanwhile, they should solve the problem of online medical insurance reimbursement, and make effort to realize the integration of online and offline medical insurance, as well as simplify the medical insurance reimbursement process.

However, the present study also has some limitations. Firstly, since some evaluation indicators of doctors involved internal data of hospitals, corresponding data could not be obtained in the research at present, so it is impossible to make practical application judgment. Secondly, the present study does not provide a face-to-face meeting for experts to discuss disagreement. Thirdly, there are a great number of indicators, and it is therefore necessary to remove those indicators with low operability in the future according to empirical research on different application scenarios.

In future studies, we will collect more diversified data to improve the evaluation index system. Meanwhile, we will establish cooperative relationships with platforms and hospitals to verify the effectiveness of the index system.

## 6. Conclusion

This is the first study to establish an online medical platform doctor evaluation system under the background of “internet + medical health” in China, and its effectiveness should be further testified through similar studies in other countries. Generally speaking, the evaluation index system constructed in this study innovatively integrate two paradigms of internet hospital proposed by the State Council under the complicated background of “internet + medical health” and the promotion and implementation of the medical and health personnel evaluation mechanism in China. In theory, this paper regards the online medical platform as an information system and applies the IS success model to the field of doctor evaluation for the first time. In order to apply the IS success model into the field of doctor evaluation more flexibly, we propose a modified DeLone and McLean IS success model, named IS success-doctor evaluation model (ISS-DE model) and defines the essential factors of ISS-DE model in detail. And this model is used as a theoretical framework to guide the article to construct the evaluation index system. Meanwhile, in the dimension of service quality, the “TFPV” model is innovatively constructed. Finally, in order to build a more comprehensive evaluation index system, indicators that are lacking in the current platform but have significance for the evaluation of doctors are prospectively included.

Realistically, the index system can help the managers of hospitals and platforms to incorporate online data when evaluating doctors and realize the integration of online and offline medical services, thereby formulating more appropriate evaluation standards for doctors. These standards can be used as a basis to assess doctors’ comprehensive quality and basic situation of doctors, providing a foundation for their work assessment and promotion.

## Data availability statement

The original contributions presented in the study are included in the article/Supplementary material, further inquiries can be directed to the corresponding author.

## Author contributions

SL and YZ: conceptualization, methodology, and resources. YZ: supervision and writing—review. SL: writing—original draft and editing, investigation, visualization, and data curation. All authors contributed to the article and approved the submitted version.
