# Supplementary material for: Designing a doctor evaluation index system for an online medical platform based on the information system success model in China
Source: Front Public Health. 2023 Oct 12;11:1185036. doi: 10.3389/fpubh.2023.1185036 (PMC10602723; doi:10.3389/fpubh.2023.1185036)
Supplement: Supplementary file 1 [file Table_1.DOCX]

Appendix 1 Interpretation of Final Index System and Evaluation Indicators

| Primary index | Secondary index | Three-level index | Index interpretation |
| --- | --- | --- | --- |
| A1  System quality | B1  Type of doctors | C1 The tier level of hospitals | Refers to tier level of hospital according to the "Hospital Classification Management Standard". According to the function of the hospital from low to high, it is divided into level 1, level 2 and level 3. Each level is divided into three levels: A, B and C. According to the scale of hospital equipment and the level of medical service, among which the third-level hospital is added with a special grade. |
|  |  | C2 Doctors’ title | The titles in the clinical system of doctor's professional title system are divided into resident, attending doctor, deputy chief doctor and chief doctor. |
|  |  | C3 Academic title | The titles in the teaching and scientific research system in the doctor's professional title system include lecturer, associate professor and professor. |
|  |  | C4 Doctors’ education | Doctors' learning experience of receiving scientific and cultural education and skills training in educational institutions. Usually refers to the highest or last degree obtained by a doctor. |
|  |  | C5 Working years | Time spent by doctors in medical work. |
|  |  | C6 Graduate school | The school where doctors graduated. |
|  |  | C7 Work-results | The doctor's achievements after completing the decisive work. |
|  |  | C8 Teaching experience | Teaching experience: the experience of doctor's theoretical teaching and clinical teaching in class.  Further education experience: the experience of a doctor being sent to a higher hospital to study.  Academic exchange experience: academic exchange refers to the experience of doctors participating in academic exchange activities.  Social post: doctors hold the post of president or chairman of societies or associations at all levels. |
|  | B2 Influence of doctors | C9 Reprint volume | The forwarding number of popular science content published by doctors. |
|  |  | C10 Number of likes | The number of likes received by doctors' answers and published popular science content. |
|  |  | C11 Collection quantity | The number of popular science content published by doctors. |
|  |  | C12 Attention number | Number of people who follow the doctor's homepage. |
|  |  | C13 Favorable rate | The proportion of favorable comments received by doctors to the total number of comments received by doctors. |
|  |  | C14 Recommendation rate | The rate at which doctors are recommended. |
|  |  | C15 Adoption rate | The proportion of doctors' answers adopted to the total number of doctors' answers. |
|  |  | C16 Number of patients | Total number of patients treated by doctors online. |
|  |  | C17 Reserved quantity | The total number of patients booking the doctor through the online platform. |
|  |  | C18 Page View | Number of visits to the doctor's homepage. |
|  |  | C19 Peer review | Peer recognition and praise to doctors. |
|  |  | C20 Recommended degree | Recommended value of the platform for doctors. |
|  |  | C21 Contribution value | The contribution of doctors in the platform. |
|  |  | C22 Is it ‘recommended’? | The platform decides whether to recommend doctors to patients according to certain criteria. |
|  |  | C23 Comprehensive score | The platform obtains the comprehensive score of doctors according to the public medical, academic and patient evaluation information. |
|  |  | C24 Doctor label | The platform displays the key information of doctors with labels to facilitate patients to quickly understand the doctor's situation. Including doctors' working years, response speed, national ranking of departments, weekly praise ranking, etc. |
|  |  | C25 Complaint | The number of complaints from patients to doctors. |
|  |  | C26 Number of questions answered | The number of patients' questions answered by doctors in the online platform Q&A section. |
|  |  | C27 Follow-up rate | Probability of follow-up of patients. |
|  |  | C28 Offline medical treatment rate | Probability of offline treatment for patients. |
| A2  Service quality | B3 Technical quality | C29 Basic theoretical knowledge of clinical medicine | Doctors have basic theoretical knowledge of clinical medicine. |
|  |  | C30 Basic pharmacological knowledge and clinical rational drug use knowledge | Doctors have basic pharmacological knowledge and clinical rational drug use knowledge. |
|  |  | C31 Judgment and interpretation of common auxiliary examination | Doctors have the correct judgment and interpretation of common auxiliary examinations. |
|  |  | C32 Mastery and application of routine diagnosis and treatment operation technology | Doctors can master and apply routine diagnosis and treatment techniques skillfully. |
|  |  | C33 Diagnosis and treatment of common and frequently-occurring diseases | Doctors have the ability to correctly diagnose and deal with common diseases and frequently-occurring diseases. |
|  |  | C34 Majors are good at diseases | The number of diseases that doctors are good at. |
|  |  | C35 Treatment experience | Doctor's history of treating diseases. |
|  | B4 Functional quality | C36 Interpersonal communication skills | Doctors have good communication skills with patients. |
|  |  | C37 Patient-centered service concept | Respect patients, make them fully informed, encourage patients to give feedback and listen to their opinions carefully. |
|  |  | C38 Abides by professional ethics and behavioral ethics | Follow the code of conduct that should be followed in medical practice activities |
|  |  | C39 Can provide confidence for patients | Establish confidence for patients to fight the disease. |
|  |  | C40 Confidentiality | Doctors keep the contents of patients' questions and their symptoms confidential. |
|  |  | C41 Service interactivity | Doctors can fully communicate with patients. |
|  |  | C42 Interactive real-time | Doctors can respond to patients' questions in time. |
|  |  | C43 Service friendliness | Doctors can be approachable and amiable. |
|  | B5 Value quality | C44 Rationality of charges | The doctor's fee is reasonable. |
|  |  | C45 The price difference for different services provided by doctors | The difference in fees between doctors providing different services is reasonable. |
|  | B6 Procedure quality | C46 Service content personalization | Give personalized responses and solutions to different patients and medical records. |
|  |  | C47 Personalized service mode | Doctors can provide more types of services. |
|  |  | C48 Service continuity | Doctors can pay regular visits to patients. |
| A3 Information quality | B7 Information content | C49 [pertinence](javascript:%20void(0)) | The relevance of information content to questions. |
|  |  | C50 Professionality | Information content is professional and formal. |
|  |  | C51 Accuracy | Fact of information. |
|  |  | C52 Objectivity | The expression of information is pure and has nothing to do with the publisher's position and situation. |
|  |  | C53 Integrity | Whether the information content is orderly, reasonable in layout, distinct in levels and clear in organization. |
|  |  | C54 comprehensibility | The information is understandable and easy to understand. |
|  |  | C55 Expression diversity | The information content is not only displayed in words, but also includes pictures, videos and voices. |
|  |  | C56 Comprehensiveness | Complete coverage of information content. |
|  |  | C57 Simplicity | Information content can use concise language. |
|  | B8 Information utility | C58 Validity | Information can solve patients' problems. |
|  |  | C59 Practicality | Practical operability of information. |
|  |  | C60 Safety | The risk of information in use. |
